# Vitamin D as an Immunomodulator: Risks with Deficiencies and Benefits of Supplementation

**DOI:** 10.3390/healthcare3020219

**Published:** 2015-04-14

**Authors:** Jason R. Goldsmith

**Affiliations:** Department of Internal Medicine, University of Michigan, 3116G Taubman Center, SPC 5368, Ann Arbor, MI 48109, USA; E-Mail: goldsmja@med.umich.edu

**Keywords:** vitamin D, immune function, immunomodulator, inflammatory bowel diseases, multiple sclerosis, rheumatoid arthritis, Treg

## Abstract

Vitamin D refers to a class of fat-soluble secosteroids often associated with their role in absorption and metabolism of minerals such as calcium and phosphate. In recent years, our understanding of vitamin D has expanded to include its role in modulating the immune system. Of particular focus are the effects of vitamin D deficiency and supplementation on patients suffering from disorders due to dysregulation of the immune system. In patients with multiple sclerosis, rheumatoid arthritis, and inflammatory bowel disease, deficiencies in vitamin D have been associated with an increased risk of disease activity. In this review, we will look at the current state of research in regards to the relationship between vitamin D and immune-dysregulation. We will focus on both the risks associated with vitamin D deficiency as well as the benefits of vitamin D supplementation.

## 1. Introduction

Vitamin D refers to a series of steroid hormones typically associated with calcium and phosphate absorption and metabolism. Vitamin D comes in two main forms, vitamin D3 (cholecalciferol) and vitamin D2 (ergocalciferol). Both forms of vitamin D occur in limited amounts in the diet and sun-dependent synthesis of vitamin D3 is the primary source in humans [1]. Once ingested, vitamins D2 and D3 are first hydroxylated in the liver at the 25-position, and then undergo further hydroxylation in the kidney to become activated. The active form of vitamin D3 is known as calcitriol, or 1,25(OH)2D. Research is mixed on whether vitamin D2 or D3 is more effective in elevating circulating 25-hydroxy vitamin D3, often used as surrogate marker of efficacy [2,3,4], although the weight of evidence is now shifting to vitamin D3 as the superior agent [5,6].

While vitamin D is prototypically associated with mineral reabsorption and metabolism, in particular playing a key role of in the metabolism of calcium and phosphate, in recent years the scientific community has come to appreciate that it plays an important role in modulating immune activity. In the 1980s, the vitamin D receptor (VDR) was found to be on located in human peripheral blood monocytes and activated B and T-cells [7,8], and further work confirmed that VDR is located in all major T-cell lineages as well as macrophage/monocytes [9]. More recently, vitamin D has been shown to be an inhibitor of dendritic cell maturation [10,11], as well as T-cell stimulatory function [11], and B-cell differentiation and proliferation [12]. It has also been found to affects T-cell differentiation, shifting polarization to preferentially favor Th2 cell development (with IL-4, IL-5, and IL-10 production) over Th1 development (INF-gamma production) [13].

Work with murine bone-marrow-derived dendritic cells (BMDCs) have demonstrated similar findings, with vitamin D exposure altering the BMDCs to promote Treg production over cytotoxic T-cells [14]. In asthmatics and in healthy patients, vitamin D has been shown to decrease the generally pro-inflammatory Th17-response [15]. Active vitamin D has also been shown to increase production of Foxp3(+) Treg *in vitro*, in a manner inhibited by IL-10 [16], through direct interaction of the VDR with the FOXP3 gene [17]. Other studies have found that active vitamin D induces IL-10 secreting Tregs that also co-express toll-like receptor (TLR9), and whose IL-10 secretory and Treg activities were down-regulated by TLR9 activation [18]. *In vitro* work has similarly found vitamin D to down-regulate INF-γ gene expression through direction modulation of gene promoter activity [19].

Additionally, vitamin D has also been found to regulate the production of anti-microbial peptides cathelicidin and defensin beta2 [20]. In addition to its anti-microbial properties, cathelicidin is an enhancer of the epithelial barrier [21], providing an additional mechanism by which vitamin D might promote homeostasis—Namely repair of damaged epithelial barriers.

The aforementioned signaling properties of vitamin D have translated into a mixed picture in various auto-immune diseases. Vitamin D has been implicated as both beneficial and harmful in asthma [22], suggesting that it plays a complex role in the inflammatory response that drives that disease state. In other diseases, such as inflammatory bowel disease (IBD), multiple sclerosis (MS), and rheumatoid arthritis (RA), vitamin D deficiency has generally been associated with an increase in the risk of the disease and/or worse disease activity, and supplementation has generally been found to be beneficial [22]. Below, we review the role of vitamin D in IBD, MS, and RA in increasing the risk of the disease and modulating disease activity, as well as the molecular signaling of vitamin D in relation to the pathophysiology of each disease state.

## 2. Inflammatory Bowel Disease

In recent years, vitamin D deficiency has been implicated in the pathogenesis of IBD. One of the initial observations is that IBD has higher incidences in more northern climates [23], where there is less sun exposure and thus generally higher levels of vitamin D deficiency [1,24]. Additionally, higher predicted vitamin D status is associated with decreased risk of Crohn’s Disease (CD) [25]. More recently, genetic analyzes have revealed a link between the vitamin D receptor (VDR) gene and IBD. Genome-wide association studies show a linkage between IBD and region of chromosome 12 that colocalizes to the VDR gene [26,27,28]. Single-nucleotide polymorphism (SNP) analysis of VDR demonstrates a strong association between one SNP, *TaqI*, and CD [29].

In terms of disease activity, several studies show an inverse relationship between IBD and vitamin D levels [30,31,32,33,34], while others have shown no association between Crohn’s Disease Activity Index (a common disease-status reporting tool) and vitamin D levels, although an inverse relationship between the anti-inflammatory cytokine IL-10 and vitamin D was noted [35]. *In vitro* assays using peripheral T cells of CD patients found that vitamin D enhanced several cytokines, including IL-10, IL-4, and IL-6, while INF-γ levels were down-regulated, demonstrating a strong immunomodulatory role for the hormone [36]. Vitamin D deficiency has also been implicated in IBD-associated colorectal cancer; decreased serum levels were found to be associated with increased risk of colorectal cancer (CRC) [37], in a concentration-dependent manner.

Much of the work studying the relationship between vitamin D and intestinal inflammation has been done in murine models. In the murine TNBS-model of colitis, vitamin D deficiency lead to worse disease activity and fibrosis as determined by collagen deposition, possibly through the TGF-β1/Smad3 pathway [38]. Similarly, in the IL-10^−/−^ model of colitis, vitamin D deficiency exacerbated disease while supplementation ameliorated disease [39,40], which correlates well with the aforementioned inverse relations between IL-10 and vitamin D seen in CD patients [35]; however, vitamin D supplementation did not reduce colonic inflammation in this model [41]. Double IL-10/VDR knockout studies demonstrated that loss of vitamin D resulted in decreased lymphocyte function leading to increased intestinal inflammation in the absence of IL-10 [42]. In a Smad3^−/−^/bacteria-driven model of murine colitis/CRC, vitamin D supplementation reduced colonic inflammation and decreased development of CRC [43]. In contrast to the generally beneficial effects of vitamin, in a hybrid IL10^−/−^/CD4+transfer/piroxicam model of colitis, vitamin D supplementation exacerbated bone mineral density degradation induced by colitis [44].

VDR has also been shown to be important in maintaining the intestinal barrier, which coincides with initial studies demonstrating that it can promote factors that enhance the epithelial barrier [20,21]. In the dextran sodium sulfate (DSS) model of acute intestinal injury, loss of the VDR receptor resulted in increased intestinal damage [45,46], while supplementation was found to be protective [47]. Loss of VDR resulted in defective autophagy function (ATG16L1 and lysozyme transcription and translation), which correlated with human intestinal IBD samples where decreased levels of VDR were associated with decreased expression of ATG16L1 [45]—A deficit recently implicated in IBD pathogenesis [48]. Furthermore, a combination of murine-DSS and *in vitro* assays also demonstrated that loss of VDR was associated with disruption in intestinal tight junctions, while vitamin D supplementation enhanced TJ integrity [46,47].

Lack of vitamin D has also been shown to alter the microbiome in mice [49]. The microbiome is known to play a critical role in IBD [50], suggesting that part of vitamin D’s effects could involve microbiome alterations, although a causal relationship has yet to be established.

Some preliminary studies have also been done looking at vitamin D as an adjunctive therapy in IBD. In a 103-patient double-blind RCT, vitamin D administration reduced relapse rates in CD by greater than 2-fold but failed to reach statistical significance (*P* = 0.06), thought to be due to insufficient power of the study [51]. In a small, open-label study of CD patients, active vitamin D (calcitriol, 1,25(OH)2D) reduced disease activity and CRP levels more than patients receiving regular vitamin D3 [52]. In another small open label study where vitamin D was supplemented to a target serum level of 40 ng/mL or a maximum supplement dose of 5000 IU/day, statistically significant reductions in disease activity were observed, although oddly no changes in systemic markers of inflammation (ESR, CRP) or cytokine levels (TNF, IL17, IL10, VEGF) were seen [53]. In a double-blind RTC, vitamin D supplementation in clinically quiescent CD patients was shown to improve fatigue, hand-grip strength, and quality of life [54].

Together, the existing body of work demonstrates that vitamin D deficiency occurs in IBD, and that supplementation can have a beneficial effect. Whether vitamin D deficiency is part of the pathogenic process of IBD remains to be determined, and larger RTCs still need to be performed to fully validate the benefit and role of vitamin D supplementation in IBD, although many clinicians currently screen IBD patients for vitamin D deficiency and treat accordingly [55]. The exact mechanism(s) by which vitamin D signaling interacts with the dysfunction underlying IBD also remains to be determined, but the literature demonstrates both immunomodulatory and epithelial-restitutive properties, suggesting that the hormone may act on multiple pathways known to be disrupted in IBD [48,56,57,58]. Another limit of this data is that it has primarily focused on CD, and not ulcerative colitis (UC), and thus the extent to which vitamin D may be beneficial in UC, outside of fending of bone mineral density loss, remains unclear and warrants further study.

## 3. Multiple Sclerosis

Like with IBD, the incidence of MS is higher in more northern latitudes where there is typically less sun exposure [59], and indeed decreased sun exposure also correlates with an increased incidence of MS [60]. A seminal nest-case control study in 2006 found that higher levels of circulating vitamin D is associated with decreased risk of developing MS [61]. Higher levels of vitamin D have also been associated with decreased disease progression [62], development of new brain lesions [63], and decreased rates of relapse in both pediatric [64] and adult [65] patients.

Recent studies suggest a possible underlying mechanism to explain the linkage between vitamin D deficiency and MS. In CNS samples from MS patients, both active lesions and normal appearing white matter had increased levels of VDR as well as the activating vitamin D hydroxylase 25(OH)D-1α-hydroxylase (CYP27B1) compared to healthy controls [66]. Given the aforementioned immunomodulatory activities of vitamin D, this suggests that in MS vitamin D may serve as an endogenous brake to inflammation, and indeed other studies support this notation. Vitamin D appears to potentiate INF-β inhibition of monocytes through up-regulation of inhibitory receptor immunoglobulin-like transcript (ILT) 3 [67]. In both healthy controls and MS patients, PBMC studies have found that vitamin D treatment decreased the proportion of effector memory T cells in favor of naïve T-cells [68]. Similar studies using PBMCs found that addition of calcitriol was able to attenuate cytokine-secretion by activated CD4+ T cells; this effect was preferentially found in PBMCs derived from MS patients compared to healthy controls [69]. Rodent studies using a model of MS have found that vitamin D administration increases Treg populations with resulting decreases in auto-reactive T-cells [70].

Recently, in a small group of patients with relapsing-remitting MS (RRMS), treatment with vitamin D increased IL10 gene expression but not TGF-β1 gene expression; this study also found a correlation between vitamin D and decreased disease activity, as measured by the EDSS (expanded disability status scale), but the sample size was small and the difference was only seen amongst patients with more severe disease [71].

Clinical trials looking at the benefits of vitamin D in MS patients unfortunately have been few and far between, with most early studies establishing that vitamin D supplementation is safe in MS patients, but were not designed with sufficient power to detect any benefit [72,73,74]. A Cochrane review in 2010 supported the view that more RTCs were needed before vitamin D supplementation could be considered an evidence-based recommendation [75], although some clinicians recommended prescribing vitamin D given its potential benefits and lack of observed harm [76].

In the last several years, there have been more RTCs supporting the use of vitamin D in MS patients, but the evidence is still not definitive. As an adjunct to interferon therapy, in a small, double-blind, placebo-controlled study vitamin D supplementation decreased MRI disease activity; although this study had issues with adequate power [77]. A small (*N* = 158) randomized, double-blind trial of the synthetic vitamin D analog alfacalcidol found broad improvements in MS patients, including decreased fatigue, improved quality of life, and decreased rates of relapse, and a higher proportion of relapse-free patients [78]. Of note, reduction in relapses became significant after 4 months of therapy and decayed after two months off of therapy [78]. Finally, the VIDMAS trial was recently announced. This multi-center, randomized, double-blinded trial plans to explore the effects of either 600 or 5000 IU/day of vitamin D3 on reducing the rate of relapse in MS patients who will concurrently be taking glatiramer acetate [79].

## 4. Rheumatoid Arthritis

The association between vitamin D deficiency and RA has remained difficult to establish. Some studies find no correlation between vitamin D deficiency and the risk of developing RA [80,81,82,83], while others have found that vitamin D deficiency increases risk of RA [84,85,86,87,88,89,90,91,92]. Some, but not all, of these studies have also found an inverse relationship between serum vitamin D levels and disease activity [86,87,89,91,92]; other studies looking at pools of RA patients have similarly shown an inverse relationship between disease severity and vitamin D levels [83,93,94]. One proposed confounder of these studies is that increased disease activity may be correlated with decreased physical activity and thus less sun exposure [95].

A recent meta-analysis demonstrated that VDR polymorphisms are associated with an increased incidence of RA, specifically the *Fok*I and *Taq*I polymorphisms [96] (the latter of which is also associated with CD) [29]. Similarly, vitamin D response elements (VDREs) are found at higher levels in RA-associated genetic loci, providing further evidence that vitamin D is involved in the development of RA.

A few mechanistic studies have been undertaken which provide some clues into how vitamin D may modulate RA. *Ex vivo* studies with murine-model and human-RA-patient derived fibroblast-like synovites (FLS) found that these vitamin D administration inhibited FLS invasion and decreased IL-1β-mediated matrix metalloprotease expression [97], suggesting the vitamin D could prevent RA-mediated bone destruction [98]. In a human synovite cell line, vitamin D was also found to decrease IL-1β-mediated l-6 and TNFβ gene expression, and increased the ratio of osteoprotegerin (OPG) to receptor activator of nuclear factor *κ*B ligand (RANKL); with enhancement of this ratio thought to limit osteoclast formation [99]. This was associated with decreased *in vitro* osteoclast formation from cell-line macrophages treated both with IL-1β and vitamin D, suggesting that vitamin D may inhibit bone loss in RA patients through blockade of inflammatory cytokine-mediated osteoclast up-regulation [99]. Similar results were seen in peripheral blood mononuclear cells taken from RA patients and treated with vitamin D in cell cultures [100]. In another study, vitamin D has deficiency has been found to be associated with increased levels of the inflammatory cytokine IL-17 and higher levels of vitamin D were associated with improved microvascular function blood flow [101].

While the data suggests that vitamin D supplementation could reduce disease activity in RA patients, little clinical work has been done to support this hypothesis. A vitamin D analog, alphacalcidiol was found years ago in a small, open-label trial to decrease disease activity in RA patients [102]. Vitamin D supplementation has also been shown to prevent bone mineral density loss in RA patients treated chronically with low-dose corticosteroids [103].

Overall, the interaction between vitamin D and RA remains poorly understood. Further studies at all levels: As a risk factor of development of RA, as a modulator of disease activity, and the molecular mechanisms of such interactions, remain to be performed.

## 5. Conclusions

Together, the evidence that vitamin D modulates immune function is quite strong. *In vitro, ex vivo,* and *in vivo* assays all suggest that vitamin D down-regulates pro-inflammatory signaling in favor of a more prototypical Treg profile. In both IBD and MS, the evidence also strongly supports the notion that deficiency in vitamin D is a disease risk factor. Disease severity for IBD and MS also appears inversely correlated to vitamin D levels, although the evidence for this observation is not as strong with more mixed results clouding whether there truly is a correlation.

Unfortunately, the benefits of vitamin D supplementation (beyond the treatment of deficiency) in inflammatory disorders remains less clear. Studies of the benefits of vitamin D face challenges unique to endogenous nutrients. Unlike many trials that compare administering a drug to a placebo where there is no drug present, vitamin D is always present in patients at some basal level. Additionally, the therapeutic zone for circulating vitamin D levels has yet to be determined [104]. These complexities have made cross-comparison of clinical trials, with different starting and supplemented levels of circulating vitamin D, difficult, and has likely contributed to the ambiguity in the field [105,106].

As it stands, the evidence supporting vitamin D supplementation ranges between Level I and Level II, with IBD having the strongest evidence and RA having the weakest evidence. Interestingly, even with the relatively lack of definitive benefit shown, the safety of such supplementation of deficient patients to normal levels of serum vitamin D is established enough to make this a category A recommendation.

While initial studies appear promising in all three diseases explored in this review: IBD, MS, and RA; large, RTCs are still needed to validate vitamin D (or analog) supplementation beyond correction of deficiency as a treatment modality for patients with these inflammatory disorders. Unfortunately, one potential barrier to this avenue of research is financial incentives for the companies that would typically undertake such work, as vitamin D is already available over the counter and while new use patents could be gained from such research, nothing would prevent off-label use of OTC vitamin D if it were found to efficacious in inflammatory diseases. Thus, further research will likely be undertaken with funding by disease-specific foundations, governmental sources, or other non-profit groups. Fortunately, the current body of literature suggests this would be a good return on investment, and hopefully will be undertaken soon.
